# Proteome-Based Analysis of Serologically Defined Tumor-Associated Antigens in Cutaneous Lymphoma

**DOI:** 10.1371/journal.pone.0008376

**Published:** 2009-12-18

**Authors:** Michael Forgber, Sylke Gellrich, Tumenjargal Sharav, Wolfram Sterry, Peter Walden

**Affiliations:** Department of Dermatology, Venerology and Allergy, Charité - Universitätsmedizin Berlin, Humboldt University, Berlin, Germany; Agency for Science, Technology and Research (A*STAR), Singapore

## Abstract

Information on specificities of serological responses against tumor cells in cutaneous lymphoma patients is relatively restricted. To advance the knowledge of serological immune responses against and to assess the scope of tumor antigenicity of cutaneous lymphoma, 1- and 2-dimensional Western blot analyses with sera from patients were combined with proteomics-based protein identification. Testing sera from 87 cutaneous lymphoma patients by 1-dimensional Western blot analysis, 64 cases of seroreactivity against lymphoma cells were found. The positive responses were relatively weak, restricted to few antigens in each case, and heterogeneous. To identify the antigens, proteins of the mycosis fungoides cell line MyLa and primary tumor cells were separated by 2-dimensional gel electrophoresis, Western-blotted and probed with heterogeneous and autologous patient sera. The antigens were identified from silver-stained replica gels by MALDI-TOF mass spectrometry. 14 different antigens were assigned and identified with this proteome-serological approach. Only one, vimentin, had been reported before, the other 13 are new antigens for cutaneous lymphomas.

## Introduction

Tumor-associated autoantigens defined by serological immune responses in cancer patients are of growing interest as potential biomarkers for disease and targets for therapy [1]–[8]. The serological antigenicity of various tumors had mostly been analyzed by serological identification of recombinant expression cloning (SEREX) with cDNA libraries of tumor tissue, tumor cells or cell lines, or of human testis cloned into λ phages and expressed by bacteria [2], [9]–[14]. The cancer-associated autoantigens identified by this approach can be categorized as differentiation antigens, cancer-testis antigens, overexpressed gene products, mutated gene products and cancer-related autoantigens. Some cancers such as systemic lymphoma, melanoma, colon carcinoma, head and neck cancer, and renal cancer have been extensively studied for serological defined antigens (http://www2.licr.org/CancerImmunomeDB/) [2], [10], [12]–[18]. On the other hand, still limited information on the antigenicity of cutaneous T cell lymphomas is available [9], [10], [19]–[24].

Cutaneous lymphomas are a heterogeneous group of lymphoproliferative disorders with primary manifestation in the skin [25], [26]. They are usually of low malignancy and evolve over extended times, often decades. At late stages, however, these cancers disseminate to lymph nodes, viscera, and bone, and in some cases develop severe hematological manifestations. High grade cutaneous lymphomas cause substantial mortality. Although, at earlier stages, the disease can efficiently be managed with a number of treatment modalities including UV radiation, cytokines and chemotherapeutics, there is no curative therapy. Most recently, antibody therapies targeting CD20, CD25 and CD52 are being tested in clinical trials with promising results [4]. Notwithstanding, long-term observations need to be awaited before conclusion can be drawn on the effectiveness of these new therapeutic instruments. Further progress in the development of these new therapies will depend on the identification of suitable target molecules. The same is true for diagnosis of cutaneous lymphomas which relies on clinical, histopathological and immunohistochemical criteria. As yet, there are no specific molecular markers for these diseases that could complement and maybe extend conventional diagnoses. Targets for therapy are not necessarily restricted to serologically detected antigens but may also include antigens recognized by T cells and identified through the analysis of secondary, i.e. T cell-dependent antibody responses.

SEREX has been successfully employed for the identification of tumor-associated antigens. However, this approach provides no information on the overall range of the serospecificities in the individual cases and occurrence of particular serospecificities in patient populations. Also, antigenicity related to posttranslational modifications is not detected by this approach. Proteome-based approaches that combine Western blot analyses of seroreactivities with mass-spectrometric protein identification can complement the molecular genetic approaches in these aspects. Proteome serology makes extensive use of the human genome sequence database for rapid identification of the serologically detected antigens. It has been applied to cancers such as renal cell carcinoma, ovarian cancer, pancreatic adenocarcinoma, prostate cancer, gastric cancer, lung squamous carcinoma and melanoma as well as infectious diseases [27]–[40]. We report here the results of a first proteome-serological analysis of the antigenicity of cutaneous lymphoma and the identification of new lymphoma-associated antigens using this technology.

## Results

### Seroreactivities to Cutaneous Lymphoma-Associated Antigens

**Figure 1:**
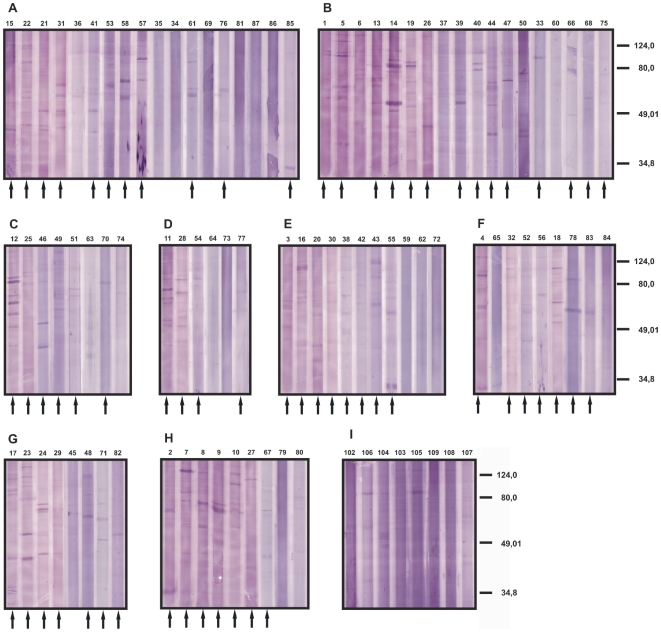
Pattern of seroreactivities of cutaneous lymphoma patients against the mycosis fungoides cell line MyLa. Total protein extract of the tumor cells were separated by SDS-PAGE, blotted onto nitrocellulose and probed with the sera of the patients or of healthy control donors. The patients were diagnosed for mycosis fungoides (**A** male, **B** female), lymphomatoid papulosis (**C**), pleomorphic cutaneous T cell lymphoma (**D**), follicle center cell lymphoma (**E**), other cutaneous T cell lymphoma (**F**): CD30+ large cell lymphoma (sera 4, 65 and 84), cytotoxic cutaneous T cell lymphomas (sera 32 and 52), small cell to medium size cell T cell lymphoma (serum 56), CD8+ epidermotropic cytotoxic cutaneous T cell lymphoma (serum 78), parapsoriasis (serum 83) and Sezary syndrome (serum) 18) and other B cell lymphomas (**G**): diffuse large cell B cell lymphoma (sera 17 and 48), large cell B cell lymphoma (serum 24), mantle cell lymphoma (sera 23, 45 and 71), leukemia (serum 82) and one not specified B cell lymphoma (serum 29), not specified cutaneous lymphomas (**H**). The lanes marked with an arrow were rated as seropositive. As controls, sera of healthy donors were used (**I**). The numbers atop of each lane represent the numbers of the sera used and correspond to the patient numbering in Table S1. This numbering is used throughout this report.

To determine the frequencies of seroreactivity against and the scope of the serospecificities for tumor cells in cutaneous lymphoma, and to identify specificities recurring in different patients we scanned the sera of 87 patients with cutaneous lymphoma by 1-dimensional Western blot analyses. All patients had been diagnosed unequivocally for cutaneous lymphoma by the clinical, histopathological, immunohistochemical and molecular genetic criteria of the EORTC/WHO classification [25] (see Table S1 for details). As protein source, the mycosis fungoides cell line MyLa was used thus focusing the search on shared antigens [42]. The cells were solubilized with SDS, solubilisates cleared of debris, separated by SDS-PAGE and blotted onto nitrocellulose. The Western blots were then probed with sera of patients with mycosis fungoides (Figure 1A and B) or other types of cutaneous lymphoma (Figure 1C–H) or control sera from healthy donors (Figure 1I). Overall, the signals were relatively weak despite high serum concentrations used for the Western blots. A large number of faint bands were detected with the sera of the healthy controls and patients alike. Notwithstanding, with 64 of the 87 patient sera relatively prominent signals were detected that were clearly stronger and in large parts different from those seen with the healthy control sera. Between 1 (e.g. sera 26 and 39) and 6 (e.g. sera 12 and 23) such prominent bands were detectable with the active sera. With two of the healthy control sera a weak band was indicated at the same migration position in the gel. The patterns of the detected antigen bands are heterogeneous with no two sera detecting the same set of antigens and seropositivity for a specific antigen band restricted to up to not more than 3 sera. The masses of the detected antigens ranged between 21 and 90 kDa with the majority of the stronger bands between 40 and 80 kDa. Comparing frequencies and patterns of seroreactivities with the diagnosed type of cutaneous lymphoma, no correlation was found, viz. seropositivity was found with similar frequencies and similarly heterogeneous specificity pattern for all the tumor entities tested, and for B and T cell lymphomas alike. In summary, a large fraction of the patients had developed prominently detectable antibody responses against antigens of the cutaneous T cell lymphoma line tested; the serospecificity patterns were heterogeneous with no antigen inducing responses in a large fraction of the patients.

### Identification of MyLa-Associated Antigens Detected by Sera of Cutaneous Lymphoma Patients

**Figure 2:**
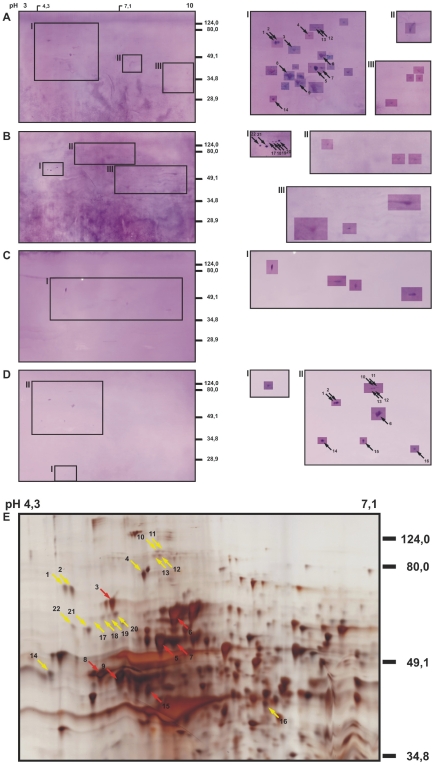
Seroreactivities of patients with cutaneous lymphoma against the mycosis fungoides cell line MyLa. Total protein extracts of MyLa cells were separated by 2-dimensional electrophoresis with an isoelectric focusing pH range of 3–10 in the first and SDS-PAGE in the second dimension, blotted onto nitrocellulose membranes and probed with the sera of the patients as listed in Table 1. Serum dilutions were 1/200. The blots **A** and **B** were probed with 8 different sera each, blots **C** and **D** with single sera. Antigens were detected in the boxed regions of the gels. The antigen signals were very weak although high concentrations of the sera were used which caused a relatively high background of the blots. To enhance the visibility of the spots, the contrast of the image areas around the spots was intensified. For every Western blot analysis **A** through **D**, the original is shown on the left and the regions with the enhanced spots for the regions indicated with Roman numerals on the right. A silver-stained gel of the MyLa proteome corresponding to the Western blots **A**–**D** is shown in **E**. The antigen spots that could be assigned to protein spots in the silver-stained gel are indicated with arrows and numbered.

**Figure 3:**
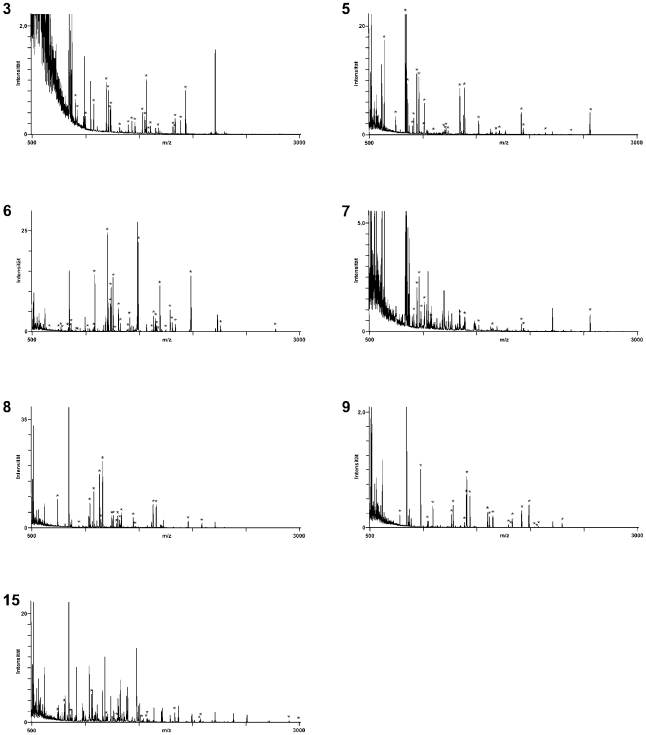
Identification of MyLa-associated antigens detected by sera of cutaneous lymphoma patients. The protein spots that could be assigned to antigen spots were picked from the gels and treated with trypsin. The resulting fragments were analyzed by mass spectrometry to identify the antigens. Seven different antigens were identified by peptide mass fingerprint analysis as BiP (spot and spectrum 3), HSP60 (spot and spectrum 5), HSP71 (spot and spectrum 6), HSP60 (spot and spectrum 7), β-tubulin (spot and spectrum 8), the β-subunit of the mt-ATP synthase (spot and spectrum 9) and TIP47, a mannose 6 phosphate receptor-binding protein (spot and spectrum 15). The peptide mass fingerprint spectra are numbered according to the antigen spots in Figure 2. The mass peaks marked with an asterisk match the theoretical spectrum of the assigned proteins. The statistics of the fingerprint analyses is summarized in Table 2.

The identification of antigens from 1-dimensional SDS-PAGE gels is complicated by the complexity of the protein mixtures at every position of the lanes and difficulties in aligning Western blots with silver-stained gels. We, therefore, separated the proteins of the MyLa tumor cell line by 2-dimensional electrophoresis with a pH gradient of 3 to 10 in the isoelectric focusing gels. Five replicas of these 2-dimensional electrophoresis gels with MyLa proteins were prepared, 4 were used for Western blot analyses (Figure 2, panels A–D) and one was silver-stained (Figure 2E). Two of the nitrocellulose filters were incubated successively with 8 different patient sera each, so to grasp as many antigens as possible and to guarantee that every Western blot displayed several antigen spots which is essential for spot pattern recognition and alignment of silver-stained proteins with Western blot spots. The sera for multiple probing were 1, 4, 5, 7, 8, 9, 11 and 12 for the blot shown with Figure 2A and 14, 18, 19, 20, 21, 22, 23 and 24 for Figure 2B. The two Western blots shown in Figure 2C and D were probed with the serum 24 and 12, respectively, alone. Altogether 16 patient sera that had shown reactivity in the 1-dimensional Western blot analyses were used for the identification of antigens in the 2-dimensional Western blots. As it was the case for the 1-dimensional Western blots, the antigen signals were weak but clearly detectable in the 2-dimensional Western blots. The western blots shown in Figure 2, panels A–D display 29, 12, 4 and 11 antigen spots, respectively (Table 1). These antigens correspond in numbers and mass distribution with the antigenicity patterns seen in the 1-dimensional Western blots (Figure 1). 27 antigens could be assigned to protein spots in the silver-stained gels (indicated with arrows in Figure 2). The antigen-protein assignments were done by first aligning gel and Western blots by their geometry and then by comparing the spot pattern in the local environments of the antigens taking spot sizes and shapes into consideration. The thus identified antigen spots were excised from the gel, destained and digested with trypsin. The resulting tryptic fragments were subjected to peptide mass fingerprint analysis by MALDI-TOF-MS and MASCOT analysis of the mass lists. 7 of the 22 assigned antigens could thereby be identified. The fingerprint mass spectra obtained for these antigens are shown in Figure 3. One antigen was identified twice in two different Western blots. In all cases the assignment of the mass spectra to the database entries is unequivocal. In the remaining cases (not shown here), the mass spectra provided insufficient information for protein identification. The antigen spots 3, 6, 8, 9 and 15 were identified as the endoplasmic chaperone BiP, the cytosolic heat shock protein HSP 71, β-tubulin, the β subunit of a mitochondrial ATP synthetase and TIP47 (a mannose 6 phosphate-binding protein), respectively. Spots 5 and 7 both were the heat shock protein HSP 60. These antigens are summarized in Table 2 together with their protein-chemical parameter, the fractions of assigned masses, sequence coverage and gene bank accession numbers.

**Table 1:** Summary of the combinations of tumor cells and sera used for mapping the serospecificities of cutaneous lymphoma patients by 2-dimensional electrophoresis and proteome analyses.

Tumor cells† *	Sera‡ *	Number of antigens#		
		total detected	assigned	identified
**Heterogeneous combinations**				
MyLa	**A**: 1, 4, 5, 7, 8, 9, 11, 12	29	12	6
MyLa	**B**: 14, 18, 19, 20, 21, 22, 23, 24	12	6	0
MyLa	**C**: 24	4	0	0
MyLa	**D**: 12	11	9	2
**Autologous combinations**				
Patient 85	85	38	13	13
Patient 86	86	12	12	9
Patient 87	87	3	0	0
Total		109	52	30

† The tumor cells used for the 2-dimensional electrophoreses were either the mycosis fungoides tumor cell line MyLa or tumor cells freshly isolated from tumor lesion of the indicated patients.

‡ The sera used to identify tumor-associated antigens by 2-dimensional Western blot analyses were obtained from patients with cutaneous lymphoma. For the heterogeneous combinations of sera and tumor cells, 8 independent sera each were grouped into two Western blot experiments. In 2 cases single sera were analyzed. The Western blots A–D are shown in Figure 2.

**Figure 4:**
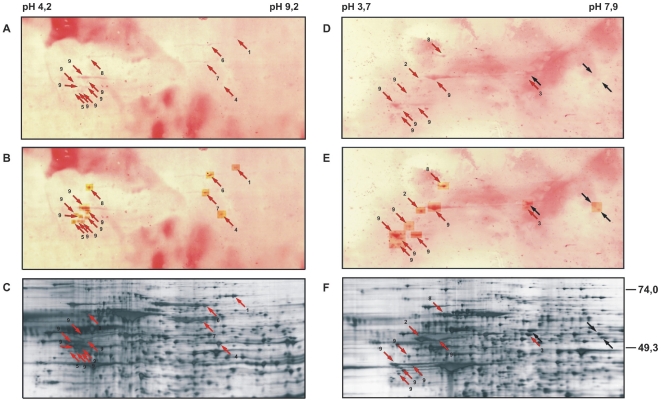
Seroreactivities of patients with mycosis fungoides against autologous tumor cells. Protein extracts of tumor cells of patient 85 (panels **A**–**C**) and patient 86 (panels **D**–**F**) were separated by 2-dimensional electrophoresis with a pH range of 3–10 for isoelectric focusing in the first and SDS-PAGE in the second dimension. The proteins were blotted onto nitrocellulose and probed with the respective sera (panels **A** and **D**) or visualized by silver staining in replica gels (panels **C** and **F**). Since the antigen reactivities are weak and their visibility further reduced by the high background which is due to the high serum concentrations that had to be used for antigen detection, the antigen spots were enhanced by image processing (panels **B** and **E**). Antigen spots that could be assigned to protein spots in the silver-stained gels are indicated with arrows and numbered.

* The numbering of the sera in this summary is the same as indicated in Figure 1 for the 1-dimensional Western blot analyses and Figures 2–4 for the 2-dimensional analyses.

# The numbers given for the antigens refer to the indicated Western blot analyses. Some antigens were identified repeatedly for different tumor cells and patients.

**Table 2:** Antigens identified for cutaneous lymphoma by proteome serology.

Spot Nr.	Antigen (gene identifier numbers)	Mw [kDa]	pI	Number of masses†		Sequence coverage†	Number of antigen spots‡
				total	matched		
	**Heterogeneous combinations**						
3	BiP (gi|6470150)	70.9	5.23	29	24	42%	1
5	HSP 60 (gi|49522865)	61.2	5.70	43	25	48%	1
6	HSP 71 (gi|32467)	70.9	5.37	85	37	50%	2
7	HSP 60 (gi|77702086)	61.2	5.70	48	14	28%	1
8	β tubulin (gi|57209813)	47.7	4.70	39	19	34%	1
9	mt-ATP Synthase, β-subunit (F1 complex) (gi|16741373)	56.5	5.26	35	22	42%	1
15	TIP47 (gi|16306789)	47.0	5.30	32	14	44%	1
	**Autologous combinations**						
1	aconitase (gi|20072188)	85.51	7.62	46	25	31%	1
2	β tubulin (gi|567053)	49.70	4.78	57	13	33%	1
3	coronin (gi|5902134)	51.00	6.25	58	18	28%	1
4	glutamate dehydrogenase (gi|20151189)	61.36	7.66	53	27	42%	1
5	keratin 16 (gi|24430192)	51.21	4.99	63	14	30%	1
6	lamin A/C isoform 1 (gi|27436946)	75.00	6.57	61	30	43%	1
7	lamin A/C isoform 2 (gi|5031875)	65.01	6.40	61	40	56%	1
8	lamin B1 (gi|5031877)	66.37	5.13	29–99	24–69-	34–43%	2
9	vimentin (gi|5030431, gi|340234)	35.02–56.55	4.82–5.06	41–95	12–51-	29–74%	13

The antigens are listed with reference to the antigen spot numbers as indicated in Figures 2 and 4, their masses and isoelectric points and the outcomes of the peptide mass fingerprints analyses.

* The spot numbers refer to the numbering given for the proteome-serological analyses for the heterogeneous and autologous combinations of tumor cells and sera, respectively.

† The numbers of peaks detected, numbers of peaks assigned and sequence coverage are the results of the peptide mass fingerprint analyses by MASCOT. In case of TIP47 a number of calculated fragments are di-, tri- or tetrapeptides with masses below 500 Dalton which set as threshold in this case.

‡ The number of antigen spots refers to all analyses reported herein. Numbers higher than 1 indicate that the same antigen was identified repeatedly in independent protein spots, some in independent electrophoresis gels, vimentin repeatedly in independent 2 gels.

### Identification of Cutaneous Lymphoma-Associated Antigens with Autologous Sera

**Figure 5:**
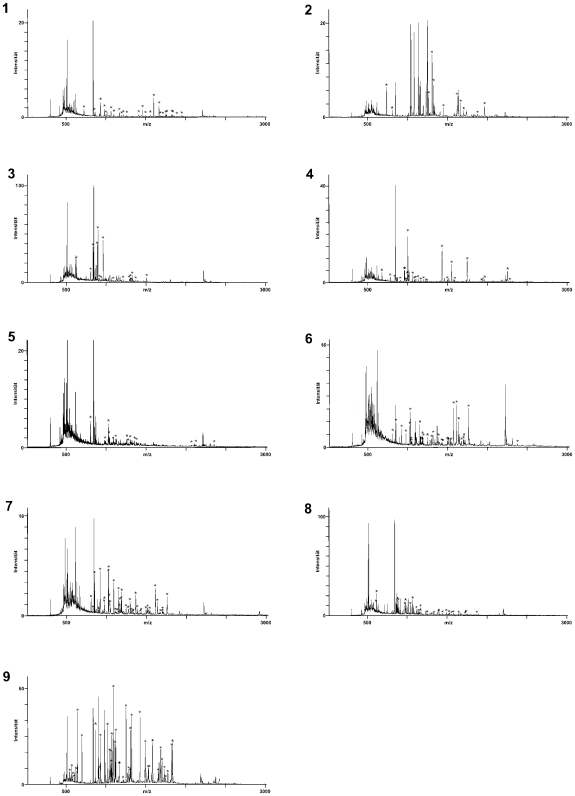
Identification of tumor-associated antigen defined by autologous sera. The protein spots assigned to antigens were picked and treated with trypsin. The peptide mass fingerprints of the resulting protein fragments were determined by mass spectrometry. Twenty-two of the assigned antigens were identified as aconitase (spot and spectrum 1), β-tubulin (spot and spectrum 2), coronin (spot and spectrum 3), glutamate dehydrogenase (spot and spectrum 4), keratin 16 (spot and spectrum 5), lamin A (spot and spectrum 6), lamin C (spot and spectrum 7), lamin B1 (spots and spectrum 8) and vimentin (spots and spectrum 9). Lamin B1 was identified in two and vimentin in 13 different spots. Only one representative spectrum is shown each. The mass peaks marked with an asterisk correspond to peptide masses which were matched to the theoretical spectrum of the identified protein. The numbers of the spectra corresponds to the numbers of the spots in Figure 4.

In three cases, patients 85, 86 and 87 (corresponding to the sera with the same numbers), tumor materials and sera were obtained so that the antigenicity of tumor cells could be analyzed with autologous sera. The tumor cells for the 2-dimensional electrophoreses were isolated from freshly excised cutaneous tumor nodules with ferromagnetic beads and antibodies with specificity for the T cell receptor (TCR) V β family of the tumor cells. The cell preparations contained more than 95% tumor cells as determined by size and TCR Vβ measured by flow cytometry (data not shown). The identities of the tumor cells were confirmed by PCRs for the tumor-specific TCR Vγ rearrangements (not shown). The protein extracts of each of the 3 tumor cell preparations were separated by two 2-dimensional electrophoresis with a pH-range of 3–10. Two replica gels for each sample were prepared, one each silver-stained and the other used for Western blot analysis with the autologous sera. In all three cases antigen spots were detected (Figure 4, Table 1). Figure 4 panels A and B, and D and E show Western blots for patients 85 and 86, respectively, and panels C and F the corresponding silver-stained gels. The antigen signals in the Western blots were very weak in both cases (Figure 4A and D) and clearly visible only after enhancement of the readings (Figure 4B and E). Nonetheless, detections were clear. 1796 proteins were counted in the silver-stained gel for the tumor cells of patient 85 (Figure 4C) and 1714 in the gel for the tumor cells of patient 86 (Figure 4F). The corresponding Western blots displayed 38 antigens for patient 85 (Figure 4A, B) and 12 antigens for patient 86 (Figure 4D, E). All 12 antigens of the tumor cells of patient 86 could be assigned to protein spots in the silver-stained gel. In the case of patient 85, 13 of the 38 detected antigens could be assigned to protein spots. For the tumor of patient 87, 3 antigens were detected, but none of them could be assigned to the corresponding silver-stained gel (data not shown). The assigned protein spots are indicated with arrows in the Western blots and silver-stained gels in Figure 4. They were picked treated with trypsin and the resulting fragments analyzed by mass spectrometry (Figure 5). The peptide mass fingerprints were processed with MASCOT for identification of the antigens. Nine of the 12 assigned antigens of the tumor cells of patient 86 and all 13 of the assigned antigens for patient 85 were identified. The respective peptide mass fingerprints are shown in Figure 5 with the masses that were matched to the theoretical spectra for the identified proteins marked with asterisks. Lamin B1 was found for both patients (spot 8). Vimentin was found 13-times in different antigen spots (spots # 9), 7-times for patient 85 and 6-times for patient 86. For these two proteins only 1 representative mass spectrum each is shown. In addition to these two, β-tubulin and coronin were found as antigens of the tumor cells of patient 86 (antigen spots 2 and 3, respectively, in Figure 4D–F). In the case of patient 85, aconitase (antigen 1), the glutamate dehydrogenase (antigen spot 4), keratin 16 (antigen spot 5), lamin A/C isoform 1 (antigen spot 6) and lamin A/C isoform 2 (antigen spot 7) were identified, all represented by a single antigen spot. The statistics for antigen identification is listed in Table 1 and the antigens, their protein-chemical characteristics, mass spectrometric sequence coverage, numbers of assigned tryptic fragments, and gene bank accession numbers together with the respective data for the MyLa-associated antigens in Table 2.

## Discussion

In all, about 160 antigens were detected in 1-dimensional Western blots with sera of 87 cutaneous lymphoma patients of whom 64 displayed a specific reactivity against at least 1 tumor-associated antigen. In average, each patients had developed specific antibody responses to 2 antigens. Some of the specific antigen bands were detected with sera of different patients but no two patients displayed the same pattern of antigenicity. In highly resolving 2-dimensional Western blots 109 antigen spots were detected with sera of 19 patients, i.e. an average of 5 to 6 per patient. Fifty-two of these antigens could be assigned to protein spots in silver-stained gels and 30 identified by the proteome-serological approach employed here. They were found to represent 14 different antigens, one, vimentin, was identified as different variants in 13 different antigen spots, three, HSP 71, HSP 60 and β-tubulin each in 2 independent gels. Two more antigens were detected with the sera of more than one patient but could not be assigned to defined spots in the silver-stained gels. In the 3 cases where the proteomes of tumor cells freshly prepared from tumor nodules could be tested with autologous sera, two antigens, lamin B1 and vimentin, were detected with two of the tumor-serum combinations with otherwise heterogeneous antigenicity patterns. Shared antigens detected in the autologous combinations of tumor cell proteomes and sera differ from those found in the heterologous combinations. Moreover, as judged from the apparent molecular masses of the antigens detected in the 1D Western blot analyses, the antigens are not shared among a majority of patients even when grouped according to diagnoses. The serological immunoreactivities against the tumor cells, thus, were heterogeneous in the 1D as well as in the 2D Western blot analyses, and in heterogeneous combinations against the mycosis fungoides cell line MyLa and in autologous combinations. This is in contrast to serological immune responses against foreign antigens as we have shown for patients with visceral leishmaniasis where a high degree of shared antigenicity is documented by matching antigen spot patterns in the 2-D Western blot system [40]. These comparisons with the high-antigenicity situations for complex infectious agents also testify to the reproducibility of the proteomics-based seroanalytics employed in the study presented here. Heterogenicity of serological anti-tumor immune responses has also been found in our studies with melanoma patients [36] and is indicated for a number of other cancers analyzed with the same technology [27], [29], [30], [32], [35], [47]–[55]. For all antigens, the signals in the Western blots were relatively weak despite high serum concentrations indicating that the magnitudes of B cell responses were low. Nonetheless, the majority of the patients with cutaneous lymphoma had mounted antibody responses against antigens of the tumor cells. Distinctively more antigens were detected in the autologous combinations of proteomes and sera than in the heterogeneous suggesting an individualized tumor cell antigenicity. All the responses were secondary, meaning IgG, responses implying repeated stimulation by the antigens and induction of MHC class II-restricted CD4^+^ helper T cells with specificity for the same antigens which is prerequisite for the Ig class switch leading to IgG-type responses.

The categories of proteins identified as antigens were to some degree different in the heterogeneous and autologous combinations of tumor cells and sera: Most antigens, particular in the autologous combinations, were structural proteins (β tubulin, coronin, keratin 16, lamin A, lamin B1, lamin C and vimentin). In the heterologeous combinations, chaperones (HSP 60, HSP 71 and BiP) were prominent. In both analyses, metabolic enzymes (aconitase, glutamate dehydrogenase, mitochondrial ATP synthetase, TIP47, a mannose-6-phosphate-binding protein) were found. Two antigens (aconitase and ATP synthetase) are mitochondrial proteins and 2 (BiP and TIP47) proteins of export pathways. Interestingly, keratin 16 was found identified as antigen in cutaneous lymphoma. Keratin 16 is part of the cytoskeleton in epithelial cells and up-regulated in wound healing, but normally not expressed by lymphoid cells. It seems ectopically expressed by the cutaneous lymphoma cells. Notable is also the antigenicity of the three nuclear intermediate filament proteins lamin A/C isoform 1, lamin A/C isoform 2 and lamin B1. The multiplicity of vimentin may indicate the presence of splice variants and, maybe, differentially phosphorylated isoforms. The detected antigens are intracellular proteins, not directly accessible to the immune system. Their immunogenicity may relate to the turn-over of the tumor cells and/or destruction of the cells induced by therapeutic interventions or tumoricidal activities of the immune system. As the proteome-based approach can also pick up posttranslational modifications, tumor-related alterations in post-transcriptional processing of proteins may also result in neo-antigenicity of otherwise normal cellular proteins.

The proteome serological approach had been used for antigen discovery for renal cell carcinoma [27], [32], [47], [48], breast cancer [49], colon carcinoma [50], prostate cancer, pancreatic adenocarcinoma [30], ovarian cancer [29], hepatocellular carcinoma [35], [51], [52], lung squamous carcinoma [53], [54], leukemia [55] and melanoma [36]. Among the antigens reported for these cancers, metabolic enzymes are most prominent and the antigenicity of these autoantigens has been related to their up-regulation in the tumor cells. Proteins of the cytoskeleton, chaperones and others were found as well but less often. While the spectrum of the different classes of antigens reported here for cutaneous lymphoma is similar, their representation is noticeably different and different for the heterologous versus the autologous combinations of sera and proteomes: Four of the 7 antigens identified with the patient sera in the MyLa proteome were chaperones and 7 of the 9 antigens found in the autologous combinations proteins of the cytoskeleton, in particular intermediate filament proteins. Similar to the situation with metabolic enzymes in other cancers, these proteins may be overexpressed and thereby of increased immunogenicity. The immunogenicity of keratin 16 may be related to its ectopic expression. Of the antigens reported herein, only vimentin had been reported as an antigen in cutaneous lymphoma before, the other 13 are new for this type of cancers [9], [10], [19]–[24]. Other antigens so far identified for cutaneous lymphoma include cTAGE and TSGA10 both of which were originally classified as cancer-testis antigens as well as a number of nuclear proteins, proteins of the transcription machinery, metabolic enzymes and others. For cTAGE, cancer testis-specific splice variations have been suggested [22]. TSGA10, despite its naming, is ubiquitously expressed and not restricted to testis [10], [56]–[58]. We had found HSP60 and aconitase also for melanoma [36], and HSP 71, vimentin and tubulin had been reported for other cancers.

The antigens found for the various cancers analyzed so far by proteome serological analyses and by SEREX show very little overlap suggesting that these two technologies probe different categories of antigens and may complement each other for the elucidation of the antigenicity of tumor cells. As for the potential use of the identified antigens in cancer immunotherapy or for cancer diagnostics, none of the antigens identified with the present study are outer membrane proteins and suited for antibody-mediated targeted therapy. However, they raise secondary antibody responses in cancer patients and, thereby, can guide the identification of epitopes for CD4^+^ MHC class II-restricted helper T cells. So far, only few such epitopes are known [59]. A large spectrum of epitopes is required for designing therapeutic vaccines for cancer immunotherapy. The heterogeneity of the antibody responses in cancer patients does not suggest single antigen-based serodiagnostics. However, protein arrays that probe antibody responses against sets of antigens identified by SEREX and proteome serology might well be of diagnostic value.

## Materials and Methods

### Tumor Cells, Cell Lines and Sera

The tumor cells were isolated from excised skin tumors by passaging through a cell sieve to remove connective tissue and MACS with antibodies for the Vβ family of the tumor cells as previously established by immunohistochemistry. By molecular genetic analysis of the TCR Vγ rearrangement, these tumor cell preparations were homogeneous (data not shown). Flow cytometry showed that by size and Vβ at least 95% of the sorted cells were tumor cells. The mycosis fungoides tumor cell line MyLa was grown in DMEM supplemented with 5% fetal calf serum, 5% newborn calf serum, 30 nM mercaptoethanol, penicillin and streptomycin at 37°C in a humidified atmosphere with 8% CO2 [41], [42]. All cells were processed for electrophoretic analysis immediately after harvest from cell cultures or collected from the cell sorter. The sera were collected from 87 patients with cutaneous lymphoma and 8 healthy donors of the Department of Dermatology, Charité. The clinical background information on the cutaneous lymphoma patients are listed in Table S1. The study and the use of materials from human subjects had been reviewed and approved by the institutional ethics committee of the Charité – Universitätsmedizin Berlin (Si. 277, September 11, 2003). The materials were obtained and used with written informed consent of the donors.

### Protein Sample Preparation

The cells were harvested by centrifugation at 1,600 x g for 10 minutes at 20°C, washed thrice with PBS and solubilized in lysis buffer (7 M urea, 2 M thiourea, 2.5% Triton X-100, 2% β-mercaptoethanol, 0.8% Pharmalyte pH 3,5–10 (LKB, Freiburg, Germany), 200 µM Pefablock® (Merck, Darmstadt), 1 µM pepstatin (SIGMA, Munich, Germany) and 10 µM leupeptin (SIGMA, Munich, Germany) as adapted from Görg [43] and Chan [44] by vortexing and sonicating in an ice-cooled water bath for 10 minutes. The cell extracts were then incubated for one hour at room temperature (RT) with 4,000 U/ml benzonase (Merck, Darmstadt, Germany) to degrade nucleic acids and centrifuged at 350,000 x g for 15 minutes at 15°C. The supernatants were collected, incubated once again with Benzonase for 10 minutes at RT and cleared by ultracentrifugation as before.

### Two-Dimensional Gel Electrophoresis (2DE)

Isoelectric focusing (IEF) was done in immobilized pH-gradient gel strips (IPG strips 180×3 mm; Pharmacia, Freiburg) with a pH range of 3 to 10 [43], [44]. Approximately 100 µg protein, i.e. the extract of 1×10^6^ cells, in 350 µl solubilization buffer were applied per IPG strip and loaded overnight at RT by in-gel re-swelling under silicon oil in nitrogen and water saturated atmosphere to prevent oxidation of the protein and drying of the gel strips. The loaded IPG strips were rinsed, mounted on a cooled ceramic plate and connected with the electrodes via wetted paper bridges to the electrodes. The IEF was run at 20°C under silicon oil in a nitrogen- and water-saturated atmosphere. The electric settings were 0.15 mA per IPG strip and voltage stepwise increased: 18 h 50 V, 1 h 150 V, 2 h 300 V, 1 h 600 V, 6.5 h 3,500 V and 3 h 5,000 V for a total of 40,000 Vh for pH 3–10 IPG strips. After the run, the IPG-strips were stored at −20°C. For the second dimension, IPG strips were thawed, rinsed with de-ionized water and equilibrated to SDS-PAGE sample buffer for 15 minutes (6 M urea, 30% glycerol, 2% sodium dodecylsulfate (SDS), Tris pH 6.8 and bromophenol blue, 1% dithiothreitol (DTT)) followed by 15 minutes in the same buffer with 4% iodoacetamide instead of DTT. The equilibrated IPG-strips were rinsed with de-ionized water and placed gel-side to gel-side onto the 4.8% acryl amide, 0.13% bisacryl amide stacking gel of a horizontal SDS polyacrylamide gel with a 12.3% acryl amide, 0.34% bisacryl amide separation gel. The settings for the runs were 1,000 V, 40 W and 20 mA for 2–3 h for transfer of the proteins from the IPG strip into the SDS polyacrylamide gel, and 1,000 V, 40 W and 40 mA for the separation until the running front reached the anodic end of the gel.

### Protein Staining

The gels were stained with the high sensitivity silver staining method by Blum and colleagues [45]. Briefly, SDS-PAGE gels were fixed in 40% Methanol, 10% acetic acid for one hour. Then, the gels were washed three times for 20 minutes in de-ionized water, sensitized for one minute with 0.02% sodium thiosulfate, washed thrice for 20 seconds in water and incubated for 20 minutes in silver staining solution (0.2% silver nitrate, 0.0074% formaldehyde). After three washes for 20 seconds in water, gels were incubated in developing solution (6% sodium carbonate, 0.00015% formaldehyde, 0.0004% sodium thiosulfate) until protein spots became visible. Reactions were stopped with 0.025% EDTA in water.

### Western Blot

Proteins from unstained SDS-PAGE were transferred to nitrocellulose membranes (Schleicher & Schüll, Dassel, Germany) by semi-dry blotting for 2 hours at 400 mA. Free binding sites on the membranes were blocked with 5% low-fat milk powder in Tris-buffered saline (TBS) for one hour at room temperature or overnight at 4°C. After blocking, the membranes were incubated with patient sera at a 200-fold dilution for one hour at room temperature, washed thrice for 10 minutes with TBS and incubated for 30 min with alkaline phosphatase-labelled anti human IgG (Anti-Human Ig-AP, Fab fragment; Boehringer Mannheim) at a 5,000-fold dilution. After washing three times for 10 minutes with TBS, the membranes were equilibrated to developing buffer (100 mM NaCl, 100 mM Tris-HCl, pH 9.5) and developed in the dark with 100 µl BCIP and 100 µl NBT in 100 ml developing buffer until antigen spots became visible. The reactions were stopped with water. For 1-dimensional Western blots, the proteins were separated by SDS-PAGE, 12% acryl amide, 0.8% bisacryl amide and blotted for 1 h with 60 mA as above.

### Alignment of Western Blot and Silver-Stained Protein Spots

For unequivocal alignment of Western blot and silver-stained spots, first, gels and blots were marked with protein spots from a pipette tip at cardinal points and aligned according to these spots. Second, adjustment of the alignments was done according to marker spots of the electrophoretically separated proteins identified on the blots after Ponceau S staining. Suitable marker proteins had previously been determined by partial blotting. Third, fine-adjustment of the alignments was done according to the spot pattern in the local environment of the antigens taking spot sizes and shapes into consideration. This approach, thus, combines automated pattern recognition with control by visual editing.

### In-Gel Proteolytic Fragmentation of the Proteins

The protein spots were excised manually with spot picker and de-stained as by Gharadaghi and colleagues [46] with 50 µl of Farmer's reducing solution (15 mM potassium ferricyanide and 50 mM sodium thiosulfate, both dissolved in water) and washed three times for 5–10 minutes with 150 µl water. Afterwards gel spots were soaked in acetonitrile and dried in vacuum. Gel pieces were re-swollen in 7.5 µl of 5 mM ammonium bicarbonate with 75 ng of modified porcine trypsin (sequencing grade, modified; Promega; Madison, USA) to fragment the protein. After 10 minutes, 7.5 µl of 5 mM ammonium bicarbonate were added and the solution with the gel pieces incubated for at least 4 hours at 37°C. For MS analysis, 1.5 µl of the aqueous supernatants were mixed with 1 µl of 2,5-dihydroxybenzoic acid (DHB) (SIGMA, Munich, Germany) (5 mg/ml water) on MALDI targets (MTP AnchorChip 600/384, Bruker Daltonik, Bremen) and air-dried.

### Mass Spectrometry

Mass spectrometric measurements were done with a Reflex IV MALDI-TOF mass spectrometer (MS; Bruker Daltonik, Bremen) in reflector mode at an acceleration voltage of 20 kV. The MS was calibrated either with the external standards angiotensin II (1046.5 Da), angiotensin I (1296.6 Da), bombesin (1619.8 Da), substance P (1347.7 Da), ACTH 1–17 (2093.0 Da) and ACTH 18–39 (2465.1 Da) or, as internal standards, with the autolytic trypsin fragments of 842.50 Da and 2211.10 Da. Monoisotopic peptide masses were determined. The spectra were processed by the Xmass software (Bruker Daltonik, Bremen) and the peaks annotated manually. Post-source decay (PSD) analyses were done in sets of 10 sections for the entire mass range and data were accumulated from up to 300 shots per section. The peak lists of the mass spectra were used for peptide mass fingerprint analyses with the Mascot software (Matrix Science; http:www.matrixscience.com/search_form_select.html) and profound (prowl; http://prowl.rockefeller.edu/profound_bin/WebProFound.exe) together with the NCBI sequence database.

## Supporting Information

Table S1 Listing of data of CB/TCL patients whose sera were tested
(0.04 MB PDF)
