# Supplementary material for: Proteome-Based Analysis of Serologically Defined Tumor-Associated Antigens in Cutaneous Lymphoma
Source: PLoS One. 2009 Dec 18;4(12):e8376. doi: 10.1371/journal.pone.0008376 (PMC2793029; doi:10.1371/journal.pone.0008376)
Supplement: Table S1 — Listing of data of CB/TCL patients whose sera were tested (0.04 MB PDF) [file pone.0008376.s001.pdf]

Proteome-based analysis of serologically defined tumor-associated antigens in cutaneous lymphoma

Michael Forgber, Sylke Gellrich, Tumenjargal Sharav, Wolfram Sterry and Peter Walden

Table S1: Listing of data of CB/TCL patients whose sera were tested

#	Sex	Age	Diagnosis	Grade/Stage	Treatment
1	F	66	ery MF	T4 N1 M0	PUVA
2	M	56	LyPap	low	no
3	F	75	FCCL	T1 N0 M0	no
4	M	69	MF	T1 N0 M0	no
5	F	73	MF	T0 N0 M0	PUVA
6	F	61	MF	T1 N1 M0	no
7	M	78	MF	T1 N0 M0	no
8	F	54	pleo CTCL	T1 N0 M0	no
9	F	56	MF	T1 N0 M0	no
10	F	72	SS	ery/leuk	ECP
11	M	54	LyPap	low	no
12	F	65	LyPap	low	no
13	F	82	CBCL	T1 N0 M0	no
14	F	77	MF	T4 N0 M0	gluco/IFN
15	M	64	MF	T2 N0 M0	no
16	M	71	FCCL	T1 N0 M0	no
17	M	72	DLBCL	low	no
18	F	83	SS	ery/leuk	gluco
19	F	55	MF	T2 N0 M0	no
20	F	30	FCCL	low	no
21	M	49	MF	T0 N3 M0	no
22	M	75	MF	T2 N0 M0	PUVA
23	M	70	MZL	T1 N0 M0	no
24	F	78	lc CBCL	T1 N2 M0	no
25	M	43	LyPap	T1 N0 M0	no
26	F	21	LyPap/MF	low	no
27	M	63	LyPap	low	no
28	M	67	pleo CTCL	low	no
29	M	39	CBCL	T1 N0 M0	no
30	M	81	FCCL	T2 N0 M0	no
31	M	49	LyPap/MF	T1 N0 M0	MTX
32	F	56	cytotox CTCL	T1 N0 M0	no
33	F	56	MF	T1 N0 M0	no
34	M	61	MF	T2 N0 M0	no
35	M	55	MF	ecz	no
36	M	60	MF	T2 N0 M0	PUVA
37	F	72	MF	T1 N0 M0	no
38	M	51	FCCL	T1 N0 M0	no
39	F	83	MF	T2 N0 M0	MTX
40	F	79	MF	T2 N1 M0	gluco
41	M	68	LyPap/MF	T2 N0 M0	MTX
42	M	56	FCCL	T1 N0 M0	no
43	F	62	FCCL	T1 N0 M0	no
44	F	66	MF	T2 N0 M0	no

#	Sex	Age	Diagnosis	Grade/Stage	Treatment
45	M	73	MF	T1 N0 M0	no
46	M	16	LyPap	T2 N0 M0	no
47	F	70	gra MF	T1 N1 M0	no
48	M	83	DLBCL leg	plaque	no
49	M	68	LyPap	T1 N0 M0	no
50	F	75	MF	T2 N0 M0	no
51	F	63	LyPap	low	no
52	F	77	cytotox CTCL	T1 N0 M0	no
53	M	76	MF	T2 N2 M0	PUVA
54	F	70	pleo CTCL	T1 N1 M0	no
55	M	67	FCCL	T2 N1 M0	no
56	F	66	pleo CTCL	T1 N0 M0	no
57	M	34	MF	T2 N0 M0	no
58	M	75	MF	T2 N0 M0	gluco
59	M	38	FCCL	T2 N0 M0	IFN
60	F	88	MF	plaque	gluco/PUV
61	M	56	MF	T1 N0 M0	no
62	M	38	FCCL	T1 N0 M0	no
63	M	77	LyPap	T1 N0 M0	no
64	F	66	pleo CTCL	T1 N0 M0	no
65	M	66	CD30$^+$ lc CL	T1 N0 M0	no
66	F	52	MF	ecz	UVB
67	M	88	MF	ecz	no
68	F	70	MF	T1 N0 M0	gluco
69	M	68	MF	ecz	no
70	M	64	LyPap	ecz	gluco
71	M	72	MCL	T1 N0 M0	IFN
72	M	66	FCCL	T1 N0 M0	no
73	M	69	pleo CTCL	T1 N0 M0	no
74	M	38	LyPap	T2 N0 M0	MTX
75	F	74	MF	T1 N0 M0	no
76	M	59	MF	T2 N0 M0	no
77	M	59	pleo CTCL	T1 N0 M0	no
78	F	80	SS	leuk	no
79	M	68	MF	T2 N1 M0	no
80	M	75	MF	plaque	no
81	M	75	MF	T2 N0 M0	no
82	M	33	leuk MF	Leuk/plaques	no
83	M	79	CTCL	T2 N0 M0	no
84	M	91	CBCL	T2 N0 M0	no
85	M	80	MF	T2 N0 M0	no
86	M	58	MF	T1 N0 M0	no
87	M	76	MF	T2 N0 M0	no

CBCL	cutaneous B cell lymphoma
CD30$^+$ lc CL	CD30 positive large cell cutaneous lymphhoma
CTCL	cutaneous T cell lymphoma
cytotox CL	cytotoxic cutaneous lymphoma
DLBCL	diffuse large B cell lymphoma
ery MF	erythrodermic MF
FCCL	follicle center cell lymphoma
leuk MF	leukemic MF
LyPap	lymphomatoid papulosis
MCL	mantel cell lymphoma
MF	Mycosis fungoides
pleo CTCL	pleomorphic CTCL
SS	Sezary syndrom

ecz	eczema stage MF
ery	erythrodermic MF
leuk	leukemia
low	low grade cutaneous lymphomas
plaque	plaque stage MF

EPC	extracorporal photophoresis
glyco	glucocorticoids
IFN	inferferon α
MTX	methotraxate
no	no treatment at the time serum was prepared
PUVA	psoralen-sensitized UV A treatment
UVB	UV B treatment
